# Relationships of serum cabozantinib concentration with tumor response and adverse events in treatment of advanced renal cell carcinoma: observational exploratory prospective study

**DOI:** 10.1007/s10147-026-03112-0

**Published:** 2026-07-04

**Authors:** Yutaro Doke, Satoshi Noda, Takuto Kusaba, Tetsuya Yoshida, Daiki Hira, Yoshito Ikeda, Susumu Kageyama, Tomohiro Terada, Shin-ya Morita

**Affiliations:** 1 Department of Pharmacotherapeutics, Shiga University of Medical Science, Seta Tsukinowa-cho, Otsu, Shiga 520-2192 Japan; 2 College of Pharmaceutical Sciences, Ritsumeikan University, 1-1-1 Noji-Higashi, Kusatsu, Shiga 525-8577 Japan; 3 Department of Urology, Shiga University of Medical Science, Otsu, Shiga 520-2192 Japan; 4 Department of Clinical Pharmacology and Therapeutics, Kyoto University Hospital, Sakyo-ku, Kyoto, 606-8507 Japan

**Keywords:** Serum cabozantinib concentration, Renal cell carcinoma, Tumor shrinkage, Adverse event, Hand-foot syndrome, Hypertension

## Abstract

**Background:**

Severe adverse events frequently occur in patients treated with cabozantinib in routine clinical care, and suboptimal responses to cabozantinib are seen in certain patients. In this prospective and observational clinical study, we investigated cabozantinib concentration–response and concentration-toxicity relationships.

**Patients and methods:**

We examined twelve patients with renal cell carcinoma treated with cabozantinib monotherapy between March 2021 and March 2024. We assessed the association of serum trough cabozantinib concentrations with the best tumor shrinkage rates. We also evaluated the association between trough cabozantinib concentrations and occurrence of adverse events.

**Results:**

There was a significant positive correlation between the concentrations of cabozantinib and the best tumor shrinkage rates (*R* = 0.64,* p* = 0.026). The trough cabozantinib concentration was significantly lower in patients without adverse events than patients with grade ≥ 1 adverse events, such as increased aspartate aminotransferase (*p* = 0.003), hand-foot syndrome (HFS) (*p* = 0.025), increased alanine aminotransferase (*p* = 0.008), hoarseness (*p* = 0.026), and dysgeusia (*p* < 0.001). Furthermore, there were significant differences in the trough cabozantinib concentrations between patients with grade ≤ 1 and patients with grade ≥ 2 adverse events, including hypertension (*p* = 0.019) and HFS (*p* = 0.038).

**Conclusion:**

In patients with renal cell carcinoma, serum cabozantinib concentrations were correlated with maximum tumor shrinkage rates in a clinical setting. This study also suggested that the occurrence of adverse events, such as HFS and hypertension, is associated with the serum concentrations of cabozantinib.

**Supplementary Information:**

The online version contains supplementary material available at 10.1007/s10147-026-03112-0.

## Introduction

Cabozantinib is an orally administered inhibitor of receptor tyrosine kinases (TKI), including mesenchymal-epithelial transition factor (MET), vascular endothelial growth factor receptors (VEGFRs) 1, 2, and 3, and AXL, which are implicated in tumor growth, angiogenesis, and metastatic progression of cancer [1]. A pivotal phase III trial (METEOR) has shown that cabozantinib extends both progression-free survival (PFS) and overall survival (OS) compared with everolimus in patients with unresectable or metastatic renal cell carcinoma (RCC) progressing after treatment with an angiogenesis inhibitor [2]. Based on these results, cabozantinib has been approved for second-line or later therapy in the United States and the European Union. However, in the METEOR trial, 71% of patients experienced grade 3 or 4 side effects, and 62% of patients reduced the cabozantinib dose due to its toxicity [3]. In a phase II clinical trial, the standard dose of cabozantinib was 60 mg/day, but, due to side effects, 91% of patients received reduced doses of cabozantinib with a median relative dose intensity of 43% in Japanese patients with metastatic RCC [4]. However, predictive markers for the toxicity of cabozantinib are unknown. Concerning efficacy, in the METEOR trial, 14% of patients showed progressive disease (PD) as the best overall response [2]. Therefore, it is essential to identify an indicator predicting the occurrence of side effects and the tumor response of cabozantinib in patients with RCC and to improve the safety and effectiveness of cabozantinib.

Oral TKIs are administrated at fixed doses despite substantial interpatient variability in oral bioavailability and distribution volume. Moreover, cabozantinib exhibits pronounced pharmacokinetic (PK) variability among patients [5]. Additionally, inter-individual differences in pharmacodynamic responses (efficacy and toxicity) remain unresolved issues in the use of cabozantinib. To address these issues, the analysis of blood exposure-efficacy and exposure-toxicity relationships could be useful. We have previously reported serum concentration-efficacy and -toxicity relationships for TKIs such as sunitinib and pazopanib [6, 7]. The high serum concentration of sunitinib is related to severe adverse effects, including decreased platelet count, anorexia, and fatigue [6]. Based on these data, therapeutic drug monitoring for sunitinib has been clinically implemented in patients with RCC in Japan. Tumor shrinkage with TKIs (sunitinib, sorafenib, and axitinib) has been reported as a significant predictor of OS in patients with RCC [8, 9]. Recently, Cerbone et al. have reported that patients with relatively low blood concentrations of cabozantinib experienced PD for RCC [10]. However, there are limited data on the relationship between the degree of tumor shrinkage and serum concentrations of cabozantinib. In addition, Cerbone et al. have reported that patients with intolerable grade 2 or grade 3 adverse events requiring treatment discontinuation or dose reduction have higher plasma concentrations of cabozantinib compared to patients without such adverse events [10]. Although cabozantinib is associated with a broad spectrum of adverse effects including hypertension, proteinuria, renal dysfunction, nausea, dysgeusia, and oral mucositis, it remains unclear whether cabozantinib toxicities are dependent on serum concentrations. The aim of this study was to evaluate the associations of serum trough cabozantinib concentrations with the best tumor shrinkage rates and adverse events in patients with RCC.

## Materials and methods

### Patients

This was a prospective observational study conducted at the Shiga University of Medical Science Hospital. We enrolled patients with RCC treated with cabozantinib monotherapy between March 2021 and March 2024. Eligibility criteria included patients aged ≥ 20 years with unresectable or recurrent advanced RCC. Exclusion criteria were as follows: patients aged < 20 years, pregnant women, and breastfeeding women. At the initiation of cabozantinib treatment, patients were classified as favorable, intermediate or poor risk according to the International Metastatic Renal Cell Carcinoma Database Consortium (IMDC) criteria. This study was conducted in accordance with the Declaration of Helsinki and approved by the ethics committee of Shiga University of Medical Science (R2020-203) and Ritsumeikan University (BKC-LSMH-2023–119). Informed consent was obtained from all participants prior to inclusion.

### Treatment plan

Cabozantinib was started at 60, 40 or 20 mg daily based on the treating physician’s discretion. Subsequently, dose reduction, discontinuation or dose escalation was adjusted based on adverse events or disease progression. Cabozantinib treatment was interrupted in patients who experienced grade ≥ 3 toxicity according to Common Toxicity Criteria for Adverse Effects (CTCAE) version 5.0 or intolerable grade ≥ 2 toxicity, until adequate recovery was achieved.

### Assessment of tumor response

The best tumor response was assessed using the Response Evaluation Criteria in Solid Tumors (version 1.1) [11]. Objective response rate (ORR) was defined as the percentage of patients who had a best response of complete response (CR) or partial response (PR). Disease control rate (DCR) was defined as the percentage of patients who had a best response of CR, PR, or stable disease (SD). For this efficacy assessment, we selected the response of the greatest tumor shrinkage. Cabozantinib has a long half-life of 55–102 h [12], and therefore, we evaluated the trough cabozantinib concentration at the time of achieving the greatest tumor shrinkage. Duration of treatment was defined as the time from initiation of cabozantinib treatment to discontinuation or the data cutoff date.

### Assessment of adverse events

All adverse events were graded according to the CTCAE version 5.0 and evaluated at each follow-up visit. For this assessment, we selected the worst clinically significant treatment-associated toxicities. We evaluated the trough cabozantinib concentration at the time of occurrence of the most serious adverse events. For patients with no adverse events, we evaluated the trough cabozantinib concentration at the end of the treatment period.

### Assessment of serum level of cabozantinib

After informed consent from the patients, blood samples were collected at steady-state trough concentrations of cabozantinib (days ≥ 14) before cabozantinib administration. Serum samples were taken at each follow-up visit after cabozantinib initiation. The blood samples were drawn into sterilized vacuum tubes and centrifuged at 1,700 × *g* and 4 °C for 10 min for serum separation. The serum was stored at -20 °C. Acetonitrile (1 mL) was added to 500 μL of serum with the internal standard (erlotinib) and vortexed thoroughly. After centrifugation at 11,000 × *g* for 10 min, the supernatant was evaporated at 65 °C under a nitrogen stream. Extraction recovery was determined by comparing the analytical results of the extracted serum control samples at three concentrations (low, 50 ng/mL; medium, 1,500 ng/mL; and high, 3,000 ng/mL) with those of each unextracted standard. The recovery rate, expressed as the mean ± standard deviation (coefficient of variation (CV), number of samples), for each concentration of cabozantinib was as follows: 50 ng/mL, 90.8 ± 3.0% (3.2%, n = 5); 1,500 ng/mL, 99.0 ± 1.6% (1.6%, n = 5); 3,000 ng/mL, 95.3 ± 2.4% (2.3%, n = 5).

Cabozantinib concentration was measured using high-performance liquid chromatography (HPLC) (auto-injector, SIL-20A HT; pump, LC-20AP; UV detector, SPD-20AV, Shimadzu, Kyoto, Japan). The serum sample was dissolved in 500 μL of N,N-dimethylformamide/water (1:1). The analysis sample was filtered through a 0.45-μm pore size filter and injected into the HPLC system. Chromatographic separation was performed using a Shim-pack XR-ODS (75 mm × 3.0 mm I.D.) column at 40 °C. Separation was achieved through gradient elution using a mobile phase consisting of phosphate buffer (pH 6.9) and acetonitrile. The gradient was 45% acetonitrile from 0 to 7 min, 85% acetonitrile from 7 to 9 min, and 45% acetonitrile from 9 to 12 min. The flow rate was 0.8 mL/min. UV detection was performed at 241 nm. The injection volume was 2 μL. The retention time was 2.0 min for erlotinib (internal standard) and 4.7 min for cabozantinib. The total analysis time required for each run was 12 min. Standard calibration curves reflected good assay linearity in the cabozantinib concentration range of 50–3,000 ng/mL. The lower limit of quantification for cabozantinib was 50 ng/mL. The precision of the method was evaluated by intra- and inter-day validations at concentrations of 250, 500, and 1000 ng/mL cabozantinib. This validation was verified using pooled human serum (MP Biomedicals, USA), cabozantinib free base (LC Laboratories, USA), and erlotinib, hydrochloride salt (LC Laboratories, USA). The precision of the method at each concentration was determined by comparing CVs. For intra-day precision of the assay, five sets of each serum control sample were assayed on the same day. For inter-day precision of the assay, each serum sample was assayed on 5 different days (days 1, 8, 15, 22, and 29). The CVs for intra- and inter-day assays were 0.9–2.8% and 2.9–9.4%, respectively (Supplementary Table 1). At all concentration levels, the precision was less than 10%.

### Statistical analysis

Data are expressed as the mean ± standard deviation (S.D.) or median. The correlation between the best tumor response rates and the trough cabozantinib concentrations was analyzed using Spearman’s correlation. Regarding adverse events, comparisons of trough cabozantinib concentrations between two groups were performed using Welch’s *t-t*est. All comparison tests were two-sided, and *p* < 0.05 was regarded as statistically significant. In these analyses, no adjustment for multiple comparisons was performed. All statistical analyses were performed using SPSS Ⅱ v. 22.0 (IBM, Armonk, NY, USA).

## Results

### Patient characteristics

Twelve patients with RCC receiving cabozantinib therapy were enrolled in this study. Table 1 summarizes the baseline characteristics of the 12 patients. The median age was 69 years (range 36–80 years), and the range of Eastern Cooperative Oncology Group Performance Status (ECOG PS) was 0–2. The median BMI was 22.3 kg/m^2^ (range 16.7–41.1 kg/m^2^). The histology of RCC was clear cell type in ten patients (83.3%). Based on the IMDC risk score evaluated at the cabozantinib initiation, nine patients (75%) and three patients (25%) were classified into intermediate and poor risk groups, respectively. Eight patients (66.7%) were treated with cabozantinib as third-line or later therapy. Supplementary Table 2 summarizes prior systemic therapy. The most recent prior systemic therapy was VEGFR-TKI (50%), immune checkpoint inhibitor (ICI) (41.7%) and VEGFR-TKI plus ICI (8.3%). The initial dose of cabozantinib was 60 mg (n = 2), 40 mg (n = 6), or 20 mg (n = 4). The most common metastatic site was bone (n = 6). Twelve patients (100%) received prior nephrectomy.

**Table 1:** Patient characteristics

	Total (n = 12)
Age (years), median (range)	69 (36–80)
*Gender, n (%)*	
Female	2 (16.7)
Male	10 (83.3)
BMI (kg/m^2^), median (range)	22.3 (16.7–41.1)
*ECOG performance status, n (%)*	
0	5 (41.7)
1	3 (25.0)
2	4 (33.3)
*Renal cancer histology, n (%)*	
Clear cell	10 (83.3)
Papillary	2 (16.7)
*IMDC classification*^*#*^*, n (%)*	
Favorable	0 (0)
Intermediate	9 (75.0)
Poor	3 (25.0)
*Previous treatment line, n (%)*	
0	0 (0)
1	4 (33.3)
≥ 2	8 (66.7)
*Initial dose of cabozantinib, n (%)*	
60 mg	2 (16.7)
40 mg	6 (50.0)
20 mg	4 (33.3)
*Most common metastatic sites, n (%)*	
Lung	3 (25.0)
Bone	6 (50.0)
Liver	1 (8.3)
Brain	1 (8.3)
Others	1 (8.3)
*Prior nephrectomy*, *n* (%)	
No prior nephrectomy	0 (0)
Prior nephrectomy	12 (100)

*ECOG* Eastern Cooperative Oncology Group Performance Status Scale, *IMDC* International Metastatic RCC Database Consortium. ^#^IMDC classification was evaluated at the initiation of cabozantinib treatment

### Changes in trough serum cabozantinib concentration with dose adjustment

**Fig. 1:**
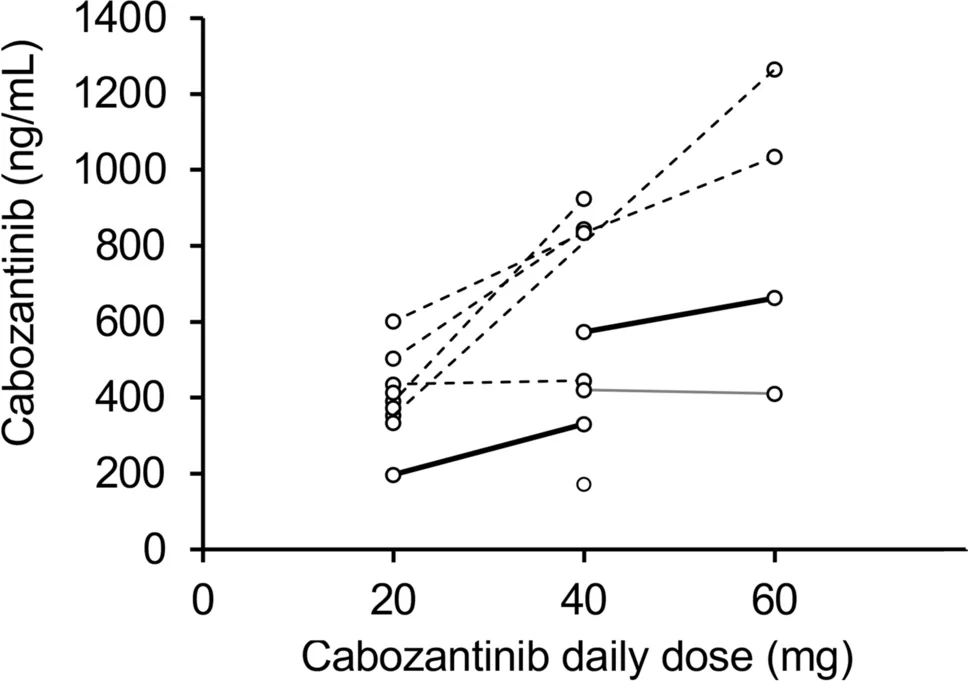
Changes in trough serum concentrations of cabozantinib with daily dose adjustment in 12 patients with RCC. The markers indicate changes in the mean trough serum concentrations of cabozantinib in the respective patients. The gray solid line represents a dose escalation (n = 1), the dotted line represents a dose reduction (n = 5). The bold solid line represents a dose escalation followed by a dose reduction (n = 2): one case started at 40 mg, escalated to 60 mg, and then reduced back to 40 mg, and another case started at 20 mg, escalated to 40 mg, and then reduced back to 20 mg

The dose of cabozantinib can be increased up to a maximum of 60 mg in one step when insufficient tumor shrinkage is observed. On the other hand, the dose of cabozantinib should be decreased by one or two steps when an adverse event of unacceptable grade 2 or higher occurs. After the dose adjustment of cabozantinib, trough cabozantinib concentrations were measured in the steady state at the next visit. Figure 1 shows the changes in the mean trough cabozantinib concentrations at the indicated doses in each patient. After the initiation of treatment with cabozantinib, the doses were escalated in 8.3% (n = 1) of patients, reduced in 41.7% (n = 5) of patients, and escalated and then reduced in 16.7% (n = 2) of patients. The mean trough cabozantinib concentrations were 400.0 ng/mL (range 193.7–601.3 ng/mL) at the 20 mg dose, 567.0 ng/mL (range 171.4–925.0 ng/mL) at the 40 mg dose, and 843.4 ng/mL (410.7–1265.9 ng/mL) at the 60 mg dose (Fig. 1). Cabozantinib concentrations differed between patients even at the same dose.

### Association of trough serum cabozantinib concentration with tumor response

**Fig. 2:**
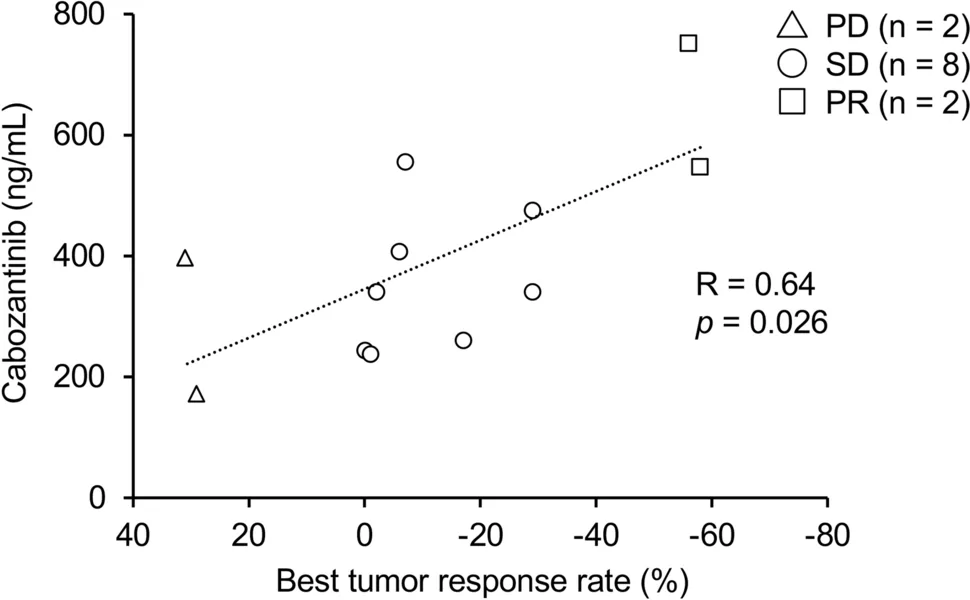
Correlation between trough serum concentrations of cabozantinib and best tumor response rates in 12 patients with RCC. The response rate was evaluated by the Response Evaluation Criteria in Solid Tumors v 1.1. R, Spearman’s rank correlation coefficient. Two, eight, and two patients exhibited progressive disease (PD, triangle), stable disease (SD, circle), and partial response (PR, square), respectively

Two (16.7%), eight (66.7%), and two (16.7%) patients had PR, SD, and PD, respectively (Table 2). The trough cabozantinib concentration (mean ± S.D.) was 357.8 ± 107.7 ng/mL (mean ± S.D., n = 8) in patients with SD (n = 8) and 416.1 ± 158.0 ng/mL in patients with disease control (DC; CR + PR + SD) (n = 10). To assess this efficacy endpoint, we examined the correlation between serum trough cabozantinib concentrations and the best percentage changes in maximum tumor diameter from baseline. A positive correlation was observed (*R* = 0.64,* p* = 0.026) (Fig. 2), suggesting that higher trough cabozantinib concentrations lead to higher rates of tumor response. Median time to best response and median duration of treatment was 2.8 months (range 1.2–8.7 months) and 4.8 months (2.5–17.3 months), respectively (Table 2).

**Table 2:** Antitumor activity of cabozantinib

	n = 12	Mean trough serum cabozantinib concentration (ng/mL)
*Best response rate, n (%)*		
CR	0	
PR	2 (16.7)	649.3
SD	8 (66.7)	357.8
PD	2 (16.7)	284.1
CR + PR (ORR)	2 (16.7)	649.3
CR + PR + SD (DCR)	10 (83.3)	416.1
Median time to best response, months (range)	2.8 (1.2–8.7)	
Median duration of treatment, months (range)	4.8 (2.5–17.3)	

*CR* complete response, *DCR* disease control rate, *ORR* objective response rate, *PD* progressive disease

*PR* partial response, *SD* stable disease

### Association of trough serum cabozantinib concentration with adverse events

**Fig. 3:**
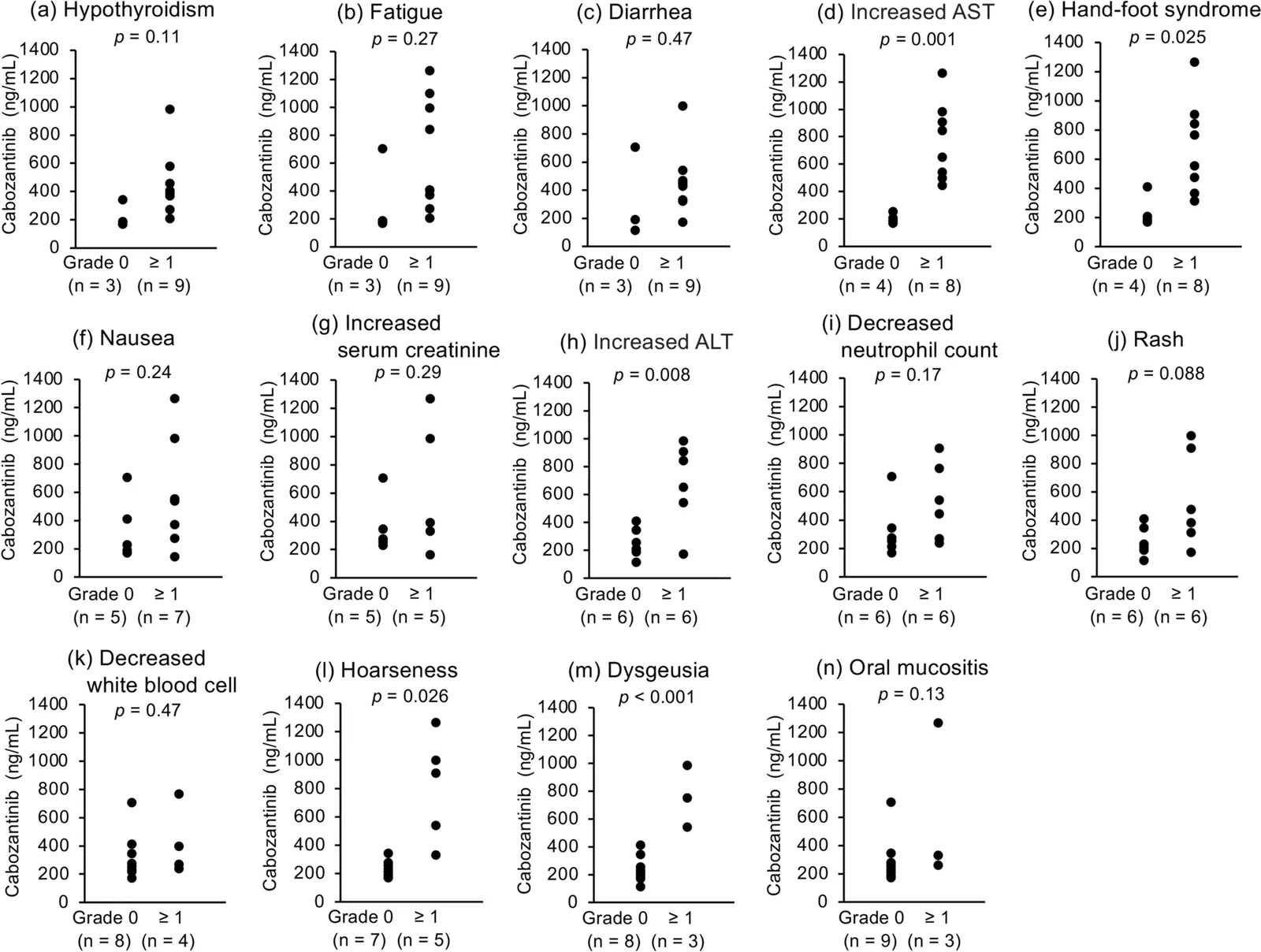
Relationships between cabozantinib serum concentration and adverse events in patients with RCC. The trough serum concentrations of cabozantinib were compared between patients without (grade 0) and with grade ≥ 1 hypothyroidism (**a**), fatigue (**b**), diarrhea (**c**), increased aspartate aminotransferase (**d**), hand-foot syndrome (**e**), nausea (**f**), increased serum creatinine (**g**), increased alanine aminotransferase (**h**), decreased neutrophil count (**i**), rash (**j**), decreased white blood cell (**k**), hoarseness (**l**), dysgeusia (**m**), and oral mucositis (**n**). All adverse events were graded using the Common Toxicity Criteria for Adverse Effects v 5.0. Two dialysis patients were excluded from the analysis of increased serum creatinine. One patient with gastrostomy was excluded from the analysis of dysgeusia. Continuous variables were compared using Welch’s t-test

**Fig. 4:**
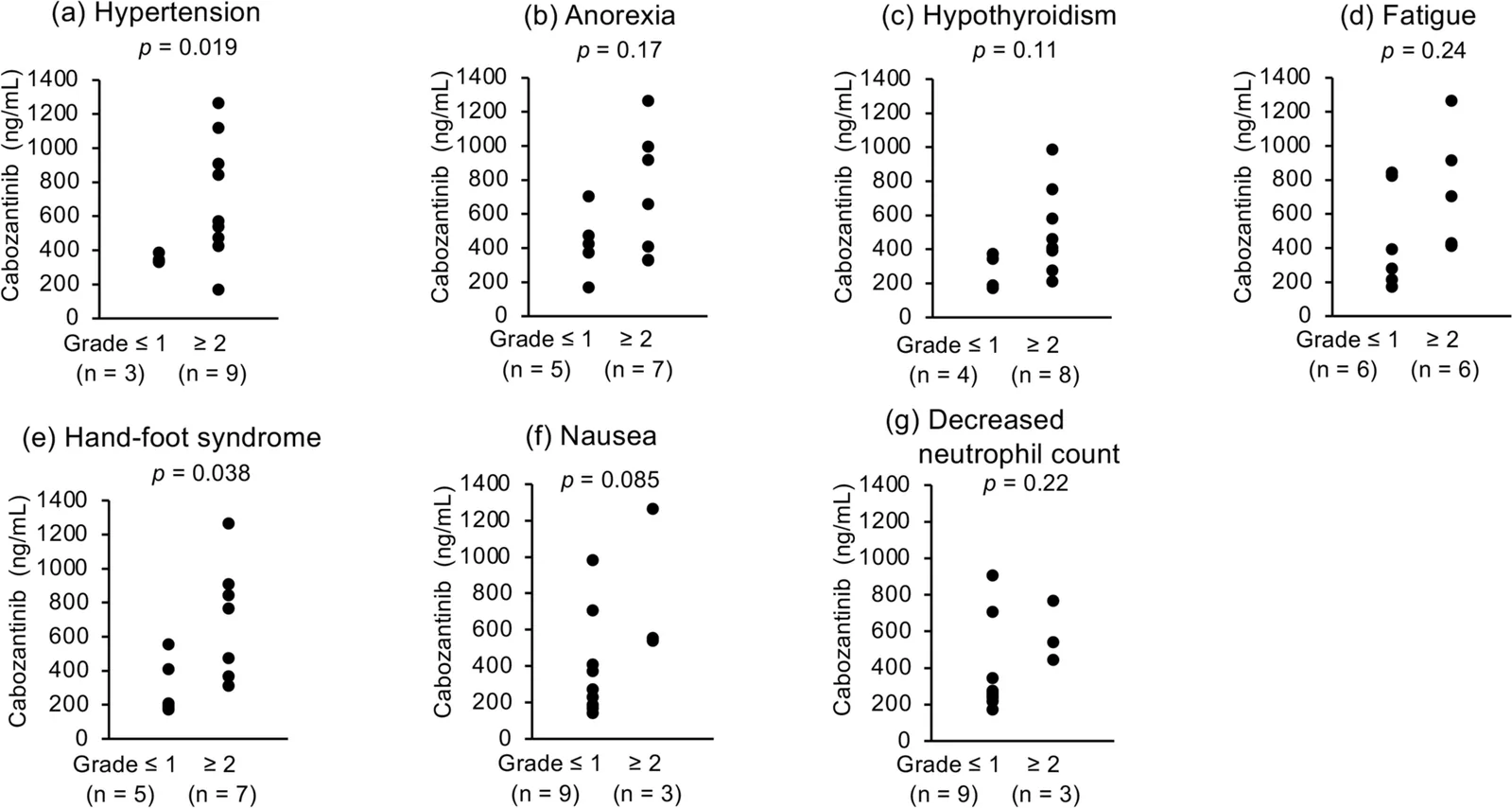
Relationships between cabozantinib serum concentration and adverse events in patients with RCC. Trough serum concentrations of cabozantinib were compared between patients with grade ≤ 1 and with grade ≥ 2 hypertension (**a**), anorexia (**b**), hypothyroidism (**c**), fatigue (**d**), hand-foot syndrome (**e**), nausea (**f**), and decreased neutrophil count (**g**). All adverse events were graded using the Common Toxicity Criteria for Adverse Effects v 5.0. Continuous variables were compared using Welch’s t-test

A summary of adverse events in patients treated with cabozantinib is shown in Table 3. Adverse events (hypertension, anorexia, hypothyroidism, fatigue, and HFS) of grade 2 or higher occurred in more than 50% of patients (Table 3). Two (16.7%), one (8.3%), one (8.3%), and one (8.3%) patients had grade 3 hypertension, increase in aspartate aminotransferase (AST), hand-foot syndrome (HFS), and increase in alanine aminotransferase (ALT), respectively (Table 3). To investigate the dependence of adverse events on serum cabozantinib concentrations, we evaluated the difference in the trough cabozantinib concentrations between patients without adverse events (grade 0) and patients with adverse events (grade ≥ 1), in which each group contained at least three patients for statistical analysis (Fig. 3). Supplementary Fig. 1 shows the comparison of serum cabozantinib concentrations for adverse events, including hypertension, anorexia, and decreased platelet count, in which the grade 0 group contained fewer than three cases. The trough cabozantinib concentration was significantly lower in patients with grade 0 compared with those with grade ≥ 1 increased AST (*p* = 0.001), HFS (*p* = 0.025), increased ALT (*p* = 0.008), hoarseness (*p* = 0.026), and dysgeusia (*p* < 0.001) (Fig. 3d, e, h, l, and m). However, the trough cabozantinib concentration did not significantly differ between patients with grade 0 and patients with grade ≥ 1 hypothyroidism, fatigue, diarrhea, nausea, increased serum creatinine, decreased neutrophil count, rash, decreased white blood cell count, and oral mucositis (Fig. 3a, c, d, f, g, i, j, k, and n). Furthermore, we compared the trough cabozantinib concentration between patients with grade ≤ 1 and patients with grade ≥ 2, in which each group contained three or more patients (Fig. 4). Supplementary Fig. 2 shows the comparison of serum cabozantinib concentrations for adverse events, in which the grade ≥ 2 group contained fewer than three patients. No significant difference was observed in anorexia, hypothyroidism, fatigue, nausea, or decreased neutrophil count (Fig. 4b, c, d, f, and g). In contrast, there were significant differences in trough cabozantinib concentrations between patients with grade ≤ 1 and patients with grade ≥ 2 hypertension (*p* = 0.019) and HFS (*p* = 0.038) (Fig. 4a and e).

**Table 3:** Occurrence of adverse events in 12 RCC patients during treatment with cabozantinib

Adverse event (n = 12)	Grade 0	Grade 1	Grade 2	Grade 3
Hypertension	1 (8.3%)	2 (16.7%)	7 (58.3%)	2 (16.7%)
Anorexia	2 (16.7%)	3 (25.0%)	7 (58.3%)	0 (0.0%)
Decreased platelet count	2 (16.7%)	8 (66.7%)	2 (16.7%)	0 (0.0%)
Hypothyroidism	3 (25.0%)	1 (8.3%)	8 (66.7%)	0 (0.0%)
Fatigue	3 (25.0%)	3 (25.0%)	6 (50.0%)	0 (0.0%)
Diarrhea	3 (25.0%)	7 (58.3%)	2 (16.7%)	0 (0.0%)
Increased AST	4 (33.3%)	7 (58.3%)	0 (0.0%)	1 (8.3%)
Hand-foot syndrome	4 (33.3%)	1 (8.3%)	6 (50.0%)	1 (8.3%)
Nausea	5 (41.7%)	4 (33.3%)	3 (25.0%)	0 (0.0%)
Increased serum creatinine^#^	5 (50.0%)	3 (30.0%)	2 (20.0%)	0 (0.0%)
Increased ALT	6 (50.0%)	5 (41.7%)	0 (0.0%)	1 (8.3%)
Decreased neutrophil count	6 (50.0%)	3 (25.0%)	3 (25.0%)	0 (0.0%)
Rash	6 (50.0%)	5 (41.7%)	1 (8.3%)	0 (0.0%)
Decreased white blood cell	8 (66.7%)	2 (16.7%)	2 (16.7%)	0 (0.0%)
Hoarseness	7 (58.3%)	4 (33.3%)	1 (8.3%)	0 (0.0%)
Dysgeusia^§^	8 (72.7%)	1 (9.1%)	2 (18.2%)	0 (0.0%)
Oral Mucositis	9 (75.0%)	3 (25.0%)	0 (0.0%)	0 (0.0%)

^#^n = 10, two dialysis patients were excluded. ^§^n = 11, one patient with gastrostomy was excluded

*ALT* alanine aminotransferase, *AST* aspartate aminotransferase, *RCC* renal cell carcinoma

## Discussion

This study showed a positive correlation between serum cabozantinib concentrations and best tumor response rates in patients with RCC in a clinical setting (Fig. 2). Our data showed that the mean trough cabozantinib concentration in patients who achieved SD or better at their best response was 416.1 ng/mL (Table 2). In a previous PK study, Cerbone et al. have reported that the cutoff value of the trough cabozantinib concentration to achieve SD or better was 536.8 ng/mL in patients with RCC [10]. However, unlike our analysis focusing on trough concentrations measured at the time of best response, the study by Cerbone et al. has reported the trough cabozantinib concentrations in the PD group measured when PD occurs after achieving the maximum tumor reduction (CR, PR, or SD) [10]. On the other hand, Blanchet et al. have reported a threshold value of 336 ng/mL to be associated with prolonged PFS in patients with RCC treated with cabozantinib 20 mg daily in an analysis based on simulated trough concentrations [13].

Cabozantinib often induces adverse events, such as hypertension, anorexia, hypothyroidism, fatigue, diarrhea, and HFS. Our study indicated that the frequency and severity of adverse events induced by cabozantinib were similar to those observed in the phase III METEOR trial [3]. Cerbone et al. have reported that patients experiencing intolerable grade 2 or 3 adverse events have higher trough cabozantinib concentrations than other patients [10]. However, it is largely unknown whether adverse events are dependent on serum cabozantinib concentrations in a clinical setting. Therefore, in this study, we investigated the relationship between the serum cabozantinib concentration and adverse events. Our study showed that the trough concentration of cabozantinib was higher in patients with grade ≥ 1 adverse events, including increased AST, increased ALT, HFS, hoarseness, and dysgeusia, compared to those without adverse events (Fig. 3). Our study also showed that significant differences were observed in the trough cabozantinib concentrations between patients with grade ≤ 1 and grade ≥ 2 in hypertension and HFS (Fig. 4). A population pharmacokinetic-pharmacodynamic analysis of the METEOR study has reported that adverse events of grade ≥ 3 fatigue, hypertension, and diarrhea are associated with a high cabozantinib concentration in the group of 60 mg/day [14]. Recently, Maruyama et al. have reported that dysgeusia, anorexia, fatigue, and dyspepsia of grade ≥ 2 significantly correlate with high trough cabozantinib concentrations [15]. The differences among our study, the METEOR trial, and the report by Maruyama et al. are partly attributable to the timing of cabozantinib concentration measurements. In our study, serum concentrations were measured at the time when the most severe adverse events occurred, whereas, in the PPK analysis of METEOR trial, serum concentrations were measured on days 29 and 57, regardless of the timing of adverse events [14]. In contrast, the analysis by Maruyama et al. has been conducted using the maximum trough cabozantinib concentrations [15].

Our study showed a relationship between trough cabozantinib concentrations and the occurrence of HFS (Figs. 3f and 4e). A relationship of the occurrence of HFS with PK of another TKI, sunitinib, has also been observed [7, 16, 17]. The mechanism of HFS has been suggested to potentially involve the secretion of drugs through sweat glands [18, 19]. Lankheett et al. have reported a trend toward increasing amounts of sunitinib in sweat gland patches with increasing severity of HFS [20]. From these findings, the accumulation of TKIs in eccrine sweat glands in the palms and soles may lead to a direct cytotoxicity in the acral skin epithelium. Therefore, high serum cabozantinib concentrations may cause severe HFS through sweat gland secretion of cabozantinib. Based on our results, PK evaluation of cabozantinib is considered to be useful for predicting HFS occurrence.

Our findings showed that serum trough cabozantinib concentrations were associated with the occurrence of hypertension (Fig. 4a). Consistent with our study, a previous PPK analysis of cabozantinib has reported the association of cabozantinib exposure with grade 3 hypertension in thyroid cancer [21]. The common inhibitory effect of TKIs on VEGFR may be implicated in hypertension [22]. The TKI-induced hypertension is due to inhibition of the VEGF-VEGFR2 pathway associated with regulation of endothelial nitric oxide synthase (eNOS) phosphorylation, NO generation, vasoconstrictor endothelin-1 production, and Ca^2^⁺ ion concentration [22]. Therefore, changes in vascular function associated with VEGFR-VEGFR2 pathway inhibition may cause hypertension. Consequently, increased exposure to TKIs may enhance the blockade of the VEGFR receptor and worsen hypertension. A previously reported PPK model has indicated that the risk of hypertension increases with higher doses and exposure of lenvatinib, a TKI [23]. We therefore consider that cabozantinib-induced hypertension is concentration-dependent.

This study includes several limitations. This study was conducted at a single center, and the number of cases for analysis was small. In addition, a large proportion of patients initiated cabozantinib treatment at the third line or later, and only two patients started cabozantinib at the 60-mg dose. Consequently, the group with high cabozantinib exposure was small, and the rates of severe adverse events were low. We recognized that the evaluation using serum drug concentration at the time of maximum tumor shrinkage or at the onset of the most severe adverse events may introduce bias, as these time points are not standardized across patients. This approach could preferentially detect higher concentrations associated with clinical events, potentially leading to an overestimation of concentration–response relationships. Although these measurements may reflect clinically meaningful pharmacodynamic effect, alternative exposure parameters, such as average serum concentration or area under the time-concentration curve, should be considered in future studies. Therefore, our results should be interpreted with caution. Furthermore, this study involved multiple exploratory analyses, and therefore the issue of multiplicity should be considered. Further determination of the therapeutic or toxic concentration ranges of cabozantinib requires prospective, large-scale clinical trials.

## Conclusions

This study suggested a positive correlation between the serum cabozantinib concentration and the maximum tumor reduction rate in patients with RCC in a clinical setting. This study also suggested that adverse events, such as HFS and hypertension, are associated with the serum concentration of cabozantinib.

## Supplementary Information

Below is the link to the electronic supplementary material.

Supplementary file1 (PDF 159 KB)

## Data Availability

The data that support the findings of this study are available from the corresponding author upon reasonable request.
