# Supplementary material for: Relationships of serum cabozantinib concentration with tumor response and adverse events in treatment of advanced renal cell carcinoma: observational exploratory prospective study
Source: Int J Clin Oncol. 2026 Jul 4;31(9):2016–24. doi: 10.1007/s10147-026-03112-0 (PMC13506646; doi:10.1007/s10147-026-03112-0)
Supplement: Supplementary file 1 [file 10147_2026_3112_MOESM1_ESM.pdf]

Supplementary Information

Relationships of serum cabozantinib concentration with tumor response and adverse events in treatment of advanced renal cell carcinoma: observational exploratory prospective study

Yutaro Doke[1], Satoshi Noda[1,2*], Takuto Kusaba[3], Tetsuya Yoshida[3], Daiki Hira[1,2,4], Yoshito Ikeda[1], Susumu Kageyama[3], Tomohiro Terada[1,4], Shin-ya Morita[1*]

[1] Department of Pharmacotherapeutics, Shiga University of Medical Science, Otsu, Shiga 520-2192, Japan; [2] College of Pharmaceutical Sciences, Ritsumeikan University, Kusatsu, Shiga 525-8577, Japan; [3] Department of Urology, Shiga University of Medical Science, Otsu, Shiga 520-2192, Japan; [4] Department of Clinical Pharmacology and Therapeutics, Kyoto University Hospital, Sakyo-ku, Kyoto 606-8507, Japan

[*] Co-corresponding authors

Supplementary Table 1. Precision of HPLC assay for the determination of cabozantinib.

	Intra-day (n = 5)			Inter-day (n = 5)	
Added (ng/mL)	Mean ± S.D.	CV (%)	Added (ng/mL)	Mean ± S.D.	CV (%)
250	237.0 ± 6.6	2.8	250	220.4 ± 20.7	9.4
500	480.3 ± 4.3	0.9	500	463.7 ± 17.2	3.7
1000	999.4 ± 26.5	2.7	1000	992.9 ± 28.9	2.9

For the intra-day assay, five sets of each serum control sample were assayed on the same day.
For the inter-day assay, each serum sample was assayed 5 different days (day 1, 8, 15, 22 and 29).
HPLC, high-performance liquid chromatography; S.D., standard deviation; CV, coefficient of variation.
CV (%) = S.D./mean × 100

Supplementary Table 2. Summary of prior systemic therapy.

	Total (n = 12)
Number of prior systemic therapy lines, n (%)	
1	4 (33.3)
2	1 (8.3)
3	7 (58.3)
Prior therapy type, n (%) *	
VEGFR-TKI	9 (75.0)
ICI	12 (100)
mTOR inhibitor	1 (8.3)
Number of prior VEGFR-TKIs, n (%)	
0	3 (25.0)
1	3 (25.0)
2	6 (50.0)
Prior VEGFR-TKI agents, n (%)	
Sunitinib	1 (8.3)
Pazopanib	7 (58.3)
Axitinib	6 (50.0)
Prior ICI agents, n (%) *	
Nivolumab	10 (83.3)
Pembrolizumab	0
Avelumab	1 (8.3)
Ipilimumab	3 (25.0)
Combination (nivolumab plus ipilimumab)	3 (25.0)
Prior mTOR inhibitor agents, n (%) *	
Everolimus	1 (8.3)
Temsirolimus	0
Most recent prior systemic therapy, n (%)	
VEGFR-TKI	6 (50.0)
ICI	5 (41.7)
mTOR inhibitor	0
VEGFR-TKI plus ICI	1 (8.3)
Reason for discontinuation of most recent prior therapy, n (%)	
Disease progression	11 (91.7)
Adverse events	1 (8.3)

*Patients may have received more than one prior therapy and/or more than one agent within each type.

ICI, Immune checkpoint inhibitor; mTOR, mammalian target of rapamycin; TKI, tyrosine kinase inhibitor; VEGFR, vascular endothelial growth factor receptor.

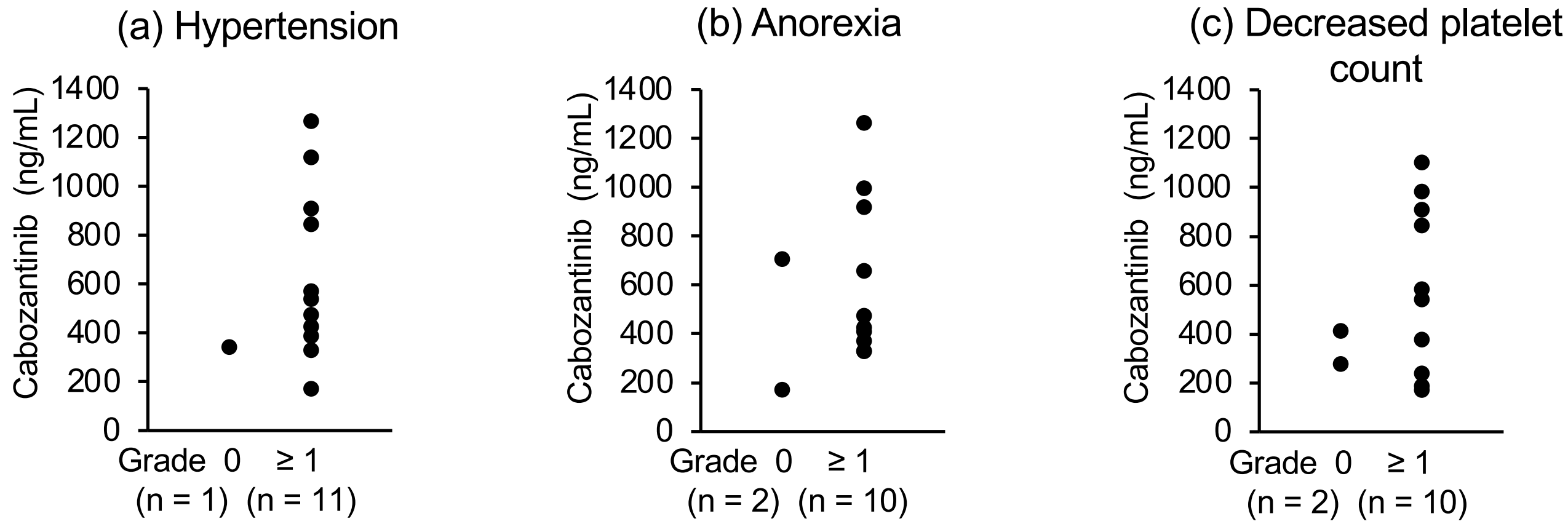

Supplementary Fig. 1. Relationships between cabozantinib serum concentration and adverse events in patients with RCC. The trough serum concentrations of cabozantinib were compared between patients without (grade 0) and with grade ≥ 1 hypertension (a), anorexia (b), and decreased platelet count (c). All adverse events were graded using the Common Toxicity Criteria for Adverse Effects v 5.0.

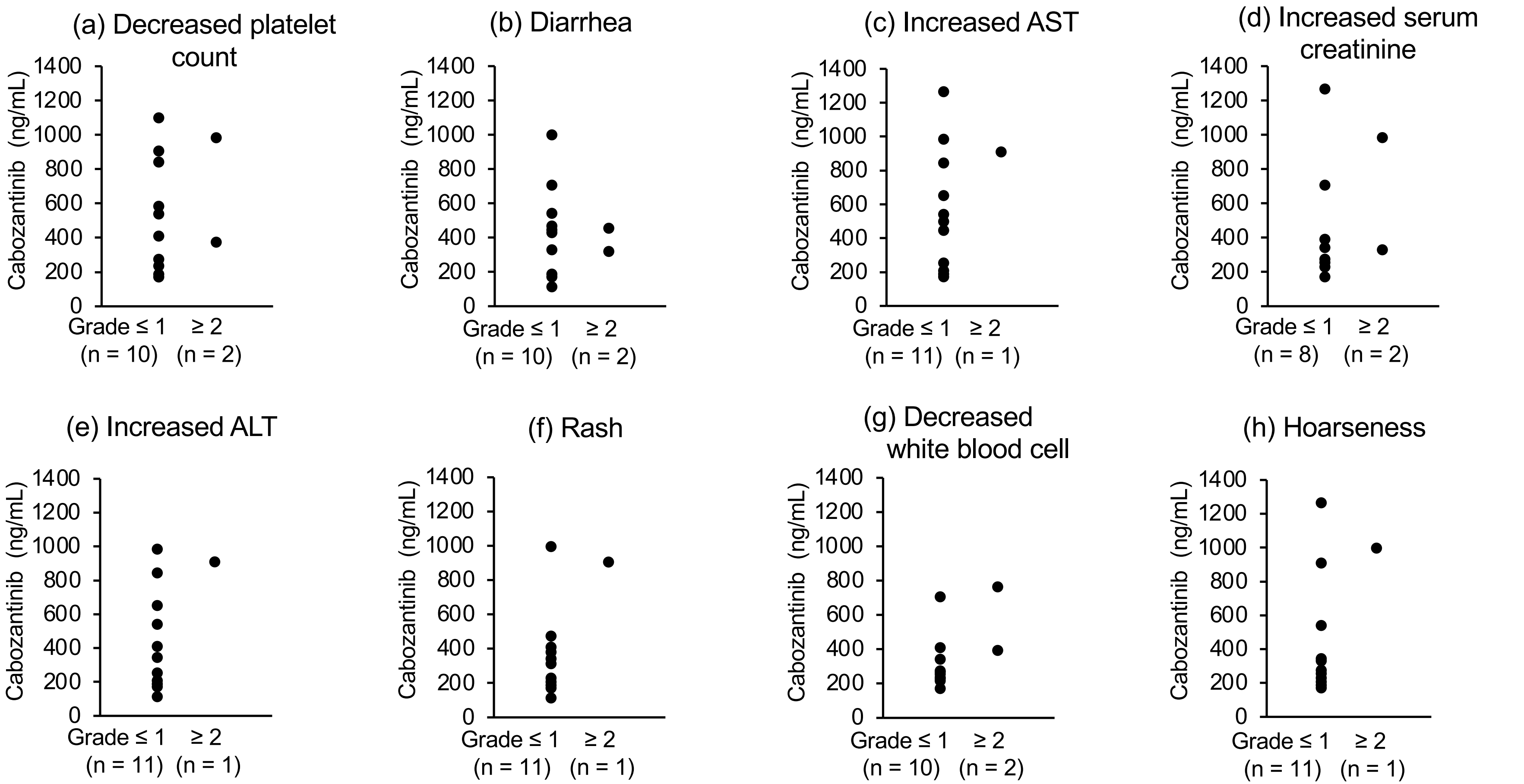

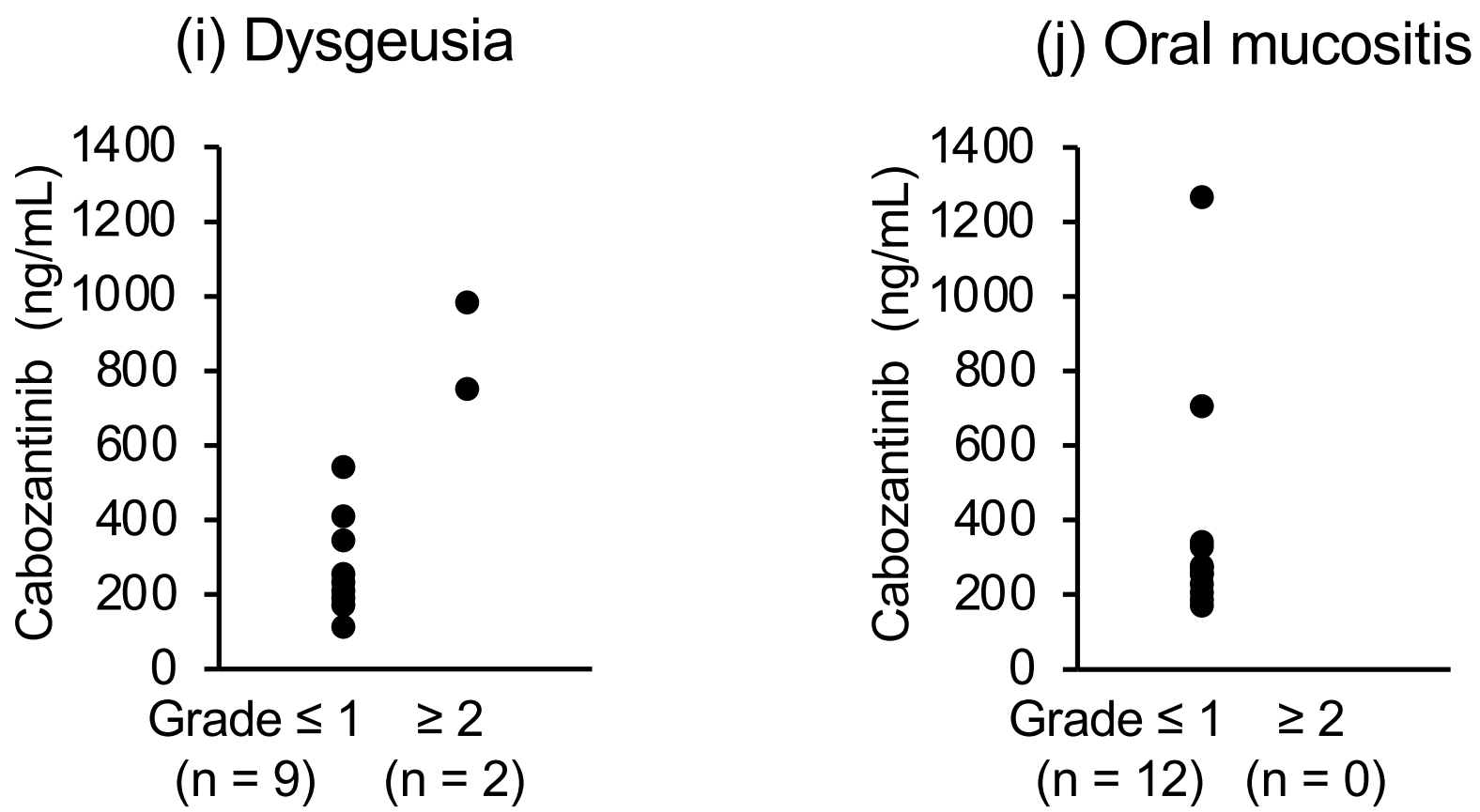

Supplementary Fig. 2. Relationships between cabozantinib serum concentration and adverse events in patients with RCC. Trough serum concentrations of cabozantinib were compared between patients with grade ≤ 1 and with grade ≥ 2 decreased platelet count (a), diarrhea (b), increased aspartate aminotransferase (c), increased serum creatinine (d), increased alanine aminotransferase (e), rash (f), decreased white blood cell (g), hoarseness (h), dysgeusia (i), and oral mucositis (j). All adverse events were graded using the Common Toxicity Criteria for Adverse Effects v 5.0. Two dialysis patients were excluded from the analysis of increased serum creatinine. One patient with gastrostomy was excluded from the analysis of dysgeusia.
